# Systematic review of cost-effectiveness analyses for combinations of prevention strategies against human papillomavirus (HPV) infection: a general trend

**DOI:** 10.1186/s12889-017-4076-3

**Published:** 2017-03-28

**Authors:** Frédéric Gervais, Kyle Dunton, Yiling Jiang, Nathalie Largeron

**Affiliations:** 1 Amaris, The Fitzpatrick Building, 188 York Way, London, N7 9AS UK; 2 Sanofi Pasteur MSD, 162 avenue Jean Jaurès, CS 50712, 69367, Lyon, Cedex 07 France

**Keywords:** Human papillomavirus, Vaccination, Public health, Cost-effectiveness

## Abstract

**Background:**

Due to the arrival of multi-valent HPV vaccines, it is more and more important to have a better understanding of the relationship between vaccination and screening programmes. This review aimed to: (1) collect published evidence on the cost-effectiveness profile of different HPV prevention strategies and, in particular, those combining vaccination with changes in screening practices; (2) explore the cost-effectiveness of alternative preventive strategies based on screening and vaccination.

**Methods:**

A systematic literature review was conducted in order to identify the relevant studies regarding the cost-effectiveness of prevention strategies against HPV infection. Analysis comparing the modelling approaches between studies was made along with an assessment of the magnitude of impact of several factors on the cost-effectiveness of different screening strategies.

**Results:**

A total of 18 papers were quantitatively summarised within the narrative. A high degree of heterogeneity was found in terms of how HPV prevention strategies have been assessed in terms of their economic and epidemiological impact, with variation in screening practice and valence of HPV vaccination found to have large implications in terms of cost-effectiveness.

**Conclusions:**

This review demonstrated synergies between screening and vaccination. New prevention strategies involving multi-valence vaccination, HPV DNA test screening, delayed commencement and frequency of screening could be implemented in the future. Strategies implemented in the future should be chosen with care, and informed knowledge of the potential impact of all possible prevention strategies. Highlighted in this review is the difficulty in assessing multiple strategies. Appropriate modelling techniques will need to be utilised to assess the most cost-effective strategies.

## Background

Cervical cancer is now the fourth most common type of cancer among women worldwide, and second most common cause of death among women aged 15 to 44 [1]. Approximately 530,000 women develop cervical cancer worldwide annually, with 85% of cases in developing countries [2, 3]. Cervical cancer is due to the human papillomavirus (HPV), a family of viruses that infect epithelial tissues of different sites [4–6]. Over 100 different types of HPV have been identified. High risk types (including 16, 18, 31 and 45) increase the risk of developing particular cancers. Low risk types 6 and 11 do not cause cervical cancer but still affect the genital area, causing 90% of genital warts. The virus also causes 90% of anal cancers, 70–75% of vaginal and vulvar cancers, and 60% of penile cancers [7, 8].

To prevent cervical cancers, screening programmes have been introduced in many countries around the world. These programmes have noticeably reduced the incidence of cervical cancer [9, 10]. Nevertheless, cervical cancer continues to be a public health problem in Europe, Australia, Canada and the USA [11–13]. In 2012, cervical cancer was estimated to cause 12,977 deaths and there were 33,354 cases of cervical cancer in the EU. In 2015, it is estimated in Canada, 1 in 152 Canadian women develop cervical cancer during her life time and 1 in 475 will die for it [11]. Similar trends follow in Australia and the USA.

The Pap smear is an essential cytological test and its introduction has led to a high reduction in cervical cancer incidence [14, 15]. Gibb and Martens, 2011, report the incidence of cervical cancer to have reduced by nearly 70% between 1955 and the mid-1980s [16]. Despite its success, the test is limited by low sensitivity, with approximately 50% of women with lesions classified as negative, and it does not detect adenocarcinoma. The low sensitivity of the test requires it to be repeated on a regular basis (every year or every 3–5 years depending on the programme) [17]. In addition, its use has plateaued or reduced in some countries leading to an HPV incidence increase [17–19].

HPV DNA testing is a recent development in the management of HPV and is much more sensitive when compared to the Pap smear [20]. The implementation of HPV DNA testing is still on-going in developing countries. Other biomarkers could enable a fully molecular-based approach to screening in the future.

Whereas screening detects diseases at an early stage (precancerous lesions) leading the treatment of these lesions prior to cancer development, vaccination prevents HPV-related disease and the burden related to treatment.

HPV vaccination has been implemented in Europe since 2007, in addition to the existing cervical cancer screening programmes. Available vaccines at that time aimed to prevent ~70% of cervical cancers and ~50% of precancerous lesions, related to HPV 16 & 18 HPV [21, 22]. The new generation vaccine (GARDASIL 9) aims to prevent ~ 90% of cervical cancers and ~80% of precancerous lesions, related to HPV 16,18,31,33,45,52,58 [23].

The vaccine was approved by the Food and Drug Administration (FDA) in 2014 for use in girls aged 9 to 26 and males aged 9 to 15. Similarly, in 2015, the Committee for Medicinal Products for Human Use (CHMP) recommended the vaccine for use in boys and girls from 9 years of age to protect against cervical, vulvar, vaginal and anal cancers, and pre-malignant cervical, vulvar, vaginal, and anal lesions and external genital warts [13]. This vaccine has the potential to further reduce the incidence of pre-cancerous lesions and cervical cancers, complementary to screening [24].

### Context and objectives

The vaccination of successive cohort of girls has the potential to reduce the average lifetime risk of developing cervical abnormalities and cervical cancer in the population; hence, the predictive value of cytology will decrease as well as the effectiveness of most screening modalities [25]. Therefore, existing screening practices will most likely evolve with regard to their frequency and strategy.

Several systematic reviews, including the recent review by Mendes et al., 2015 [25], have assessed the cost-effectiveness of different screening strategies. Other studies have evaluated the cost-effectiveness of HPV vaccination strategies and concluded that vaccination added to the existing screening programme was a cost-effective strategy [26]. However, no study has reviewed model-based cost-effectiveness studies of a potential change of screening practice in conjunction with vaccination with analysis of the sensitivity of specific parameters. In the context of the arrival of multi-valent HPV vaccines, it is more and more important to have a better understanding of the relationship between vaccination and screening programmes.

The current study aimed to review the published literature to:

- Collect published evidence on the cost-effectiveness profile of different HPV prevention strategies and, in particular, those combining vaccination with changes in screening practices
- Explore the cost-effectiveness of alternative preventive strategies based on screening and vaccination.

The study focussed on key European markets (Austria, Belgium, Czech Republic, Denmark, Finland, France, Germany, Greece, Ireland, Italy, the Netherlands, Poland, Portugal, Slovenia, Spain, Sweden, the UK, Switzerland, Norway), Australia, Canada and the USA.

## Methods

A systematic literature review was conducted in order to identify the relevant studies regarding the cost-effectiveness of prevention strategies against HPV infection. The study question was formalised according to the PICOS framework (see Appendix 1 and Appendix 2).

We searched the following electronic databases for studies published up to April 2014: MEDLINE and MEDLINE-IN-PROCESS (via Ovid, on 15th April 2014), on EMBASE (via embase.com, on 15th April 2014), and the NHS Economic Evaluation database (via cochrane.org on 15th April 2014). Search terms are included in Appendix 1. Following search completion, studies were screened and irrelevant publications excluded based on the pre-defined criteria (Appendix 1).

We included original research articles that met the following criteria:

1. Cost-effectiveness analyses based on mathematical modelling
2. Presentation of a health economic endpoint (quality-adjusted life-year or cost-effectiveness ratio) and clinical outcomes (cancers/cases avoided)

After running the searches on the electronic databases, the citations were screened by two independent reviewers, with study selection based on the pre-specified inclusion/exclusion criteria in Appendix 1. Studies of patients vaccinated against HPV infection, and with a cervical screening strategy were included. The three comparators included were vaccination against HPV infection only, an alternative cervical screening strategy only or a combination of both. The study type of interest was restricted to cost-effectiveness analyses and outcomes assessed were health economic or clinical outcomes. The exclusion criteria are detailed in Table 5 in Appendix 1. The publication selection was based on an initial review of titles and abstracts and a second review of full-texts. Quality check was conducted by the second independent reviewer and any discrepancies between the two reviewers were resolved through discussion.

Upon the completion of publication selection, relevant data were extracted according to a pre-specified template, which included authors, year of publication, country, period of analysis, mathematical model used, vaccine, price per dose and schedule, discounted rate, population, age of vaccination and catch-up, comparator, clinical outcomes considered, vaccine efficacy, duration of protection, assumptions on vaccination coverage, screening status, sensitivity analysis conducted, economic outcomes (cost per quality-adjusted life-year (QALY) and cost per life-year gained (LYG)). Data was then quantitatively summarised within a narrative review. Different scenarios on screening strategy and frequency from included studies were synthesized and plotted against changes in cost and QALY.

## Results

### Overview

**Fig. 1:**
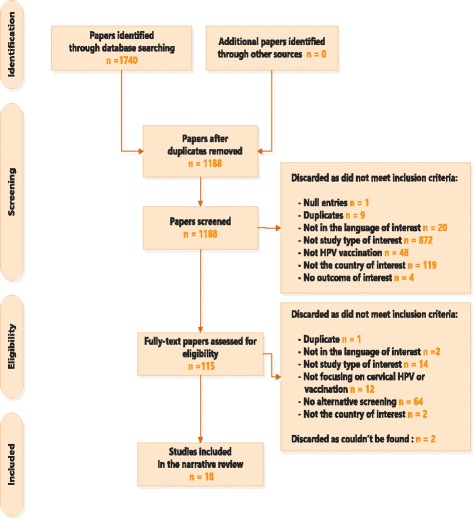
PRISMA diagram

A total of 1,188 papers were identified following removal of duplicates. Of the papers screened 115 were deemed eligible for full-text review. Following exclusion, a total of 18 papers were quantitatively summarised within the narrative review using the preferred reporting items for systematic reviews and meta-analyses (PRISMA) diagram. (see Fig. 1). Of the studies included eight focused on the US [27–34], 3 on the Netherlands [35–37], 1 on Italy [38], 1 on Australia [39], Canada [40], France [41], Norway [42], Spain [43], and Eastern Europe [44], respectively (Table 6 in Appendix 2).

Analysis comparing the modelling approaches between studies was made along with an assessment of the magnitude of impact of several factors on the cost-effectiveness of different screening strategies. Strategies which were only assessed as part of sensitivity analyses were also compared. The impacts of several factors on the cost-effectiveness of the HPV prevention strategies were assessed such as modelling approach, prevention strategies assessed, screening technique, screening frequency, age at first vaccination, screening coverage and compliance, number of vaccine valences and cross-protection, vaccine efficacy, efficacy waning effect and vaccine cost.

### Vaccination strategies

#### Vaccination programmes

Vaccination strategies varied considerably in terms of cost-effectiveness both within and between studies through both age of vaccination and number of doses received, as well as booster (Tables 1 and 2).

**Table 1:** Vaccination strategies

	Kulasingam 2003 [27]	Sanders 2003 [28]	Goldie 2004 [29]	Taira 2004 [32]	Kulasingam 2007 [39]
Vaccine considered	Bivalent	Bivalent	Bivalent	Bivalent	Bivalent
*Age of vaccination (y)*	12 (12–19)	12 (12–15)	12 (12–15)	12	12
*Catch-up*	-	-	-	-	-
*Booster*	0 (1)	10y (3y-lifetime)	- (Yes)	10y	- (1)
Vaccine price (3 doses)	$200 ($100–$600)	$300 ($100–$500)	$377 ($188–$565)	$300 ($100–$400)	AUS$345 ($300–$450)
Coverage	100% (50–100%)	-	100% (50–100%)	-	80% (70–90%)
Compliance	100%	70% (30–100%)	100%	-	-
Efficacy	90% (25–100%)	75% (0–100%)	90% (50–100%)	90% (10–90%)	100% (93–100%)
Protection duration (y)	10 (2–30)	10	-	-	Lifetime
Waning effect	-	-	No (5,10,15,20y)	Yes (No)	-
Herd Immunity	-	-	-	-	Considered in SA
	Goldhaber-Fiebert 2008 [33]	Kim 2008 [31]	Coupe, de Melker 2009 [35]	Coupe, van Ginkel 2009 [36]	Kim 2009 [30]
Vaccine considered	Bivalent	Bi/Quadrivalent	Bivalent	Bivalent	Bi/Quadrivalent
*Age of vaccination*	9	12	12	12	12
*Catch-up*	In SA	18,21,26y	-	-	-
*Booster*	-	10y	-	30y in SA	-
Vaccine price (3 doses)	$402 ($300–$900)	$360	€ 375	€ 375	$360
Coverage	25%, 75%, and 100%	75%	100%	85%	-
Compliance	-	-	100%	-	100%
Efficacy	100% (75%)	100%	95%	95% (85-90-98%)	Infection: ♀ 100%/♂ 85% Disease: ♀ 100%/♂ 90%
Protection duration	Lifetime (15y)	Lifetime	Lifetime	Lifetime	Lifetime
Waning effect	-	10 years	10 or 20 years	- (exponential decrease in efficacy of 50% during each following 20 years 10y, or 5 years)	-
Herd Immunity	- (Yes)	-	-	-	Yes
	Kim, Ortendahl 2009 [34]	Accetta 2010 [38]	Diaz 2010 [43]	Demarteau 2011 [41]	Burger 2012 [42]
Vaccine considered	Bivalent	Bivalent	Bivalent	Bivalent	Quadrivalent
*Age of vaccination*	35	11	11	12	12
*Catch-up*	-	Yes	-	up to 25y in SA	-
*Booster*	-(Yes)	-(10–20y)	No	- (1)	-
Vaccine price (3 doses)	$402 ($250–$750)	€ 309.23 (€90)	€ 312 (€150–€600)	€ 401 (€321–€482)	$489
Coverage	Not clear	100%	90% (50–100%)	-	-
Compliance	100%	-	-	-	-
Efficacy	100% (70%)	75.90% (95%)	100% (60–100%)	98% (78–100%)	-
Protection duration	Lifetime	-	Lifetime (10y)	-	-
Waning effect	-(5–10y)	-	-	-(15–20y)	-
Herd Immunity	-	-	-	-	-
	Coupe 2012 [37]	Tully 2012 [40]	Berkhof 2013 [44]		
Vaccine considered	Bivalent & multivalent	Bivalent	Bivalent		
*Age of vaccination*	Not Clear	12	12		
*Catch-up*	-	18y in SA	-		
*Booster*	-	25y in SA	-		
Vaccine price (3 doses)	-	$270 CAD	$300		
Coverage	100%	80%	70%		
Compliance	-	-	-		
Efficacy	95%	>90%	-		
Protection duration	Lifetime	Lifetime	-		
Waning effect	-(Yes)	Considered in SA (unavailable)	-		
Herd Immunity	-	Accounted	-

**Table 2:** Screening strategies

	Kulasingam 2003 [27]	Sanders 2003 [28]	Goldie 2004 [29]	Taira 2004 [32]	Kulasingam 2007 [39]
Primary screening	CC	CC	CC/LBC	CC	CC
*Compliance*	100% (50–100%)	71% (60–80%)	100%	-	By age
Triage	CC	-	HPV DNA	-	-
Cytology					
*Sens/Spe*	0.556(0.51–0.95)/ 0.957(0.80–0.97)	0.51(0.40–0.80)/ 0.97(0.95–0.98)	0.66(0.34–0.86)/ 0.97(0.88–0.99)	-	0.80(0.48–0.80)/ 0.95(0.90–0.99)
*Price*	$45 ($61–$75)	$81($61–$101)	$15–$51 ($12–75)		$58($29–$86)
HPV DNA					
*Sens/Spe*	-	-	-	-	-
*Price*			$49 ($30–$200)		
	Goldhaber-Fiebert 2008 [33]	Kim 2008 [31]	Coupe, de Melker 2009 [35]	Coupe, van Ginkel 2009 [36]	Kim 2009 [30]
Primary screening	CC/HPV DNA	CC	CC/HPV DNA	CC	CC
*Compliance*	-	-	80%	80% (20/70%)	-
Triage	HPV DNA/CC	HPV DNA (3y)	HPV DNA/CC	-	HPV DNA
Cytology					
*Sens/Spe*	0.8(0.186–0.99)/0.95(0.87–0.996)	-	-	-	-
*Price*	$30($6–$87)				
HPV DNA					
*Sens/Spe*	0.83(0.70–0.85) / 0.93(0.79–0.94)	-	0.94/1	-	-
*Price*	$55($14–$217)				
	Kim, Ortendahl 2009 [34]	Accetta 2010 [38]	Diaz 2010 [43]	Demarteau 2011 [41]	Burger 2012 [42]
Primary screening	CC/Combined	CC/HPV DNA	CC/Combined	CC	CC
*Compliance*	53% 1y, 17% 2y, 11% 3y, 15% 5y	70.90%	-	60% 3y (48%,1y-72%,5y)	100%
Triage	HPV DNA	HPV DNA/CC	HPV DNA	-	HPV DNA
Cytology					
*Sens/Spe*	0.8/0.95	0.8/0.95	0.8/0.95	0.58–0.61 (0.46–0.73)	0.8/0.95
*Price*					
HPV DNA					
*Sens/Spe*	0.93/0.93	0.96/0.94	0.88/0.93	-	1/1
*Price*					
	Coupe 2012 [37]	Tully 2012 [40]	Berkhof 2013 [44]		
Primary screening	CC/HPV DNA	CC	CC/HPV DNA		
*Compliance*	80% (40%)	-	-		
Triage	-/CC	-	-		
Cytology					
*Sens/Spe*	-	0.58–0.85/0.962–0.974	-		
*Price*	€52.80				
HPV DNA					
*Sens/Spe*	0.94/0.97	-	-/1		
*Price*	€65.6				

CC refers to the Pap smear test

Sixteen studies focused on the vaccination of pre-adolescent girls; the remaining 2 [30, 40] on catch-up programmes.

Vaccination coverage was seen to differ across studies from 70 to 100% in base case, and varied in sensitivity analyses.

#### Vaccine efficacy

Bivalent vaccine efficacy was reported to be greater than 75%, 90% and 95% versus HPV types 16/18 in 2 [27, 38], 8 [27, 29, 32, 35–37, 40, 43] and 5 [31, 33, 34, 39, 41] studies respectively, under base case. Vaccine protection duration was reported as lifelong following completion of the HPV vaccination programme in all but 2 [27, 28] studies, reporting duration as ten years.

Cross-protection against other high-risk HPV types was considered in 7 [30, 31, 33, 34, 39, 41] studies.

#### Vaccine valence

The majority of studies (17/18) focused on a bivalent (HPV16/18) or a quadrivalent vaccine (HPV6/11/16/18) (Table 3). Only one study [37] explored introduction of modelling vaccine valences of 5 to 13 (theoretical exercise only). Coupe et al., 2012 [35], concluded that an identical screening programme for vaccinated and unvaccinated women was no longer defensible if vaccinated women were protected against many high-risk HPV types by means of effective broad-spectrum vaccination. An increase in duration of screening interval was seen to lower costs but benefits as well. Broader vaccinations with valences from 5 to 13 high-risk HPV types were modelled; a 5-valent vaccine was seen to be least costly but offered fewest QALY gains, whilst a 13-valent vaccine was seen to offer greatest benefits but at greatest cost. The greater the time interval between screenings the greater the QALY gain from a vaccine of greater valence.

**Table 3:** Overview of cost-effectiveness studies included in the review

Study	Main conclusions about screening policies in post-vaccination area	Screening changes evaluated			
		Cytology/Change in age initiation	Cytology/Change in screening intervals	DNA HPV test (triage or primary test)	Others
Acetta et al. 2010 [38] (Italy)	Findings support changing the Pap screening policy to the use of HPV DNA as a primary test with Pap test triage for both vaccinated and unvaccinated women.	X	X	X	
Berkhof et al. 2013 [44] (Slovenia, Poland)	Screening with short intervals of 3 years yield only moderate benefits in term of cancer risk reduction compared to longer screening intervals. Combined vaccination and 6 to 10-yearly HPV (DNA) screening were generally cost-effective.	X	X	X	
Burger et al. 2012 [42] (Norway)	Strategies involving a switch to HPV testing for primary screening in older women are expected to be cost-effective compared with current recommendations in Norway. In the primary analysis and regardless of vaccination status, the current cytology-based screening strategy was less effective and more costly (i.e. strongly dominated) than proposed strategies that involve switching to primary HPV testing at 34 years of age.	X		X	
Coupe, de Melker et al. 2009 [35] (Netherlands)	Screening 5 times with HPV DNA (D 11,133/QALY) or 7 times with cytology (D 17,627/QALY) were scenarios with comparable costs and effects and incremental cost-effectiveness ratios below the threshold in The Netherlands (D 20,000 per QALY).	X	X	X	
Coupe, van Ginkel et al. 2009 [36] (Netherlands)	The influence of a decreasing screening compliance in vaccinated women (70% instead of 80%) has only a limited effect on the cost-effectiveness of HPV16/18 vaccination.				Changes in compliance of screening
Coupe et al. 2012 [37] (Netherlands)	In a cohort of HPV16/18 vaccinated women, four rounds of HPV DNA screening is cost-effective. One screen during lifetime remains cost-effective in addition to broad spectrum vaccination offering protection against many high-risk HPV types. In addition to broad spectrum vaccination, one screen during lifetime was cost-effective up to an 11-valent vaccine.	X	X	X	
Demarteau et al. 2011 [41] (France)	The change in screening interval was only assessed in a sensitivity analysis and had only a small effect on the ICER.		X		
Diaz et al. 2010 [43] (Spain)	After the introduction of HPV vaccination, screening will need to continue, and strategies that incorporated HPV testing are more effective and cost-effective than those with cytology alone. For vaccinated girls, 5-year organised cytology with HPV testing as triage from ages 30 to 65 costs 24,350€ per year of life saved (YLS), assuming life-long vaccine immunity against HPV-16/18 by 3 doses with 90% coverage. If high vaccination coverage among pre-adolescent girls is achieved, organized cytology screening with HPV triage starting at ages 30 to at least 65 every 4– 5 years represents the best balance between costs and benefits.	X	X	X	
Goldie et al. 2004 [29] (US)	If one imposed a minimum threshold (e.g. the reduction in cervical cancer risk over a woman’s lifetime must be at least equivalent to or greater than that in our current screening program), then the most effective strategy with an incremental cost-effectiveness ratio of less than $60,000 per QALY is one combining vaccination at age 12 with triennial conventional cytological screening beginning at age 25 years.	X	X		Liquid-based cytology is assessed
Goldhaber-Fiebert et al. 2008 [33] (US)	For both vaccinated and unvaccinated women, age-based screening by use of HPV DNA testing as a triage test for equivocal results in younger women and as a primary screening test in older women is expected to be more cost-effective than current screening recommendations.	X	X	X (HPV primary & triage)	Different screening strategy for younger and older women
Kim et al. 2008 [31] (US)	The cost-effectiveness ratios of vaccination strategies were more favourable if screening was delayed and performed at less frequent screening intervals and with more sensitive tests. The analyses concluded that cytology starting at age 25 every 3 years (with HPV DNA testing as triage), with a switch to cytology combined with HPV DNA testing starting at age 35 was similar to the base case in term of cost-effectiveness. (Base-case analysis assumes current cytology screening beginning at an average age 20–53% screened annually, 17% every 2 years, 11 every 3 years, 14% every 5 years and 5% never screened).	X	X	X	
Kim et al. 2009 [30] (US)	This study confirmed the results of Kim et al. 2008. Vaccinating preadolescent girls with cytology (HPV test for triage) every 3 years starting at age 25 and a switch to a combined cytology at age 35 had a cost-effectiveness ratio below $50,000/QALY.	X	X	X	
Kim, Ortendahl et al. 2009 [34] (US)	This US study assessed the cost-effectiveness of different strategies that combined HPV vaccination given to women older than 30 years in with different screening policies and concluded that none of the strategies were not cost-effective.		X	X	
Kulasingam et al. 2003 [27] (US)	Screening with pap tests may be delayed to a later age than currently recommended when an HPV16-18 vaccine has been given. Vaccination plus biennial screening delayed until age 24 years had the most attractive cost-effectiveness ratio ($44,889) compared with screening only beginning at age 18 years and conducted every 3 years.	X	X		
Kulasingam et al. 2007 [39] (Australia)	Vaccination of young girls and changing the screening interval and or/age of first screening would reduce costs considerably and would still be more effective than the current screening program at reducing cancer incidence and mortality.	X	X		
Sanders et al. 2003 [28] (US)	The availability of the vaccine may justify less frequent pap tests.		X		
Taira et al. 2004 [32] (US)	With a vaccine program in place, physicians must be comfortable moving to less frequent screening.		X		
Tully et al. 2012 [40] (Canada)	With a vaccine program in place for girl’s aged 12 and a coverage rate of 80%, delaying initial screening until age 21 or 25 saves costs but may cause small increases in SCC incidence and life-years lost. However, delaying the initial age of screening combined with catch-up immunization (at age 21 or 25) is predicted to save costs and reduce cancer incidence, but reduce QALYs.	X	X		

X indicates inclusion in the respective study

#### Vaccine cost

Cost of vaccination was another variable between studies with bivalent and quadrivalent 3-dose vaccine cost varying from €147.00–402.00 and €264.00–360.00, respectively.

Costs were seen to have an important effect on the ICER of optimal strategies and significantly affect the cost-effectiveness result of vaccination strategies. Demarteau et al., 2011 [41] reported a change in vaccine cost to significantly alter the ICER for vaccination of 12 year old girls. Taira et al., 2004 [32] and Kulasingam et al., 2007 [39] reported a change in vaccine cost to have a significant impact on the ICER, with a decrease in cost seen to have greater effect than an increase.

### Overview of screening approaches considered

Various HPV prevention strategies were assessed in the included studies (Table 7 in Appendix 2). All studies included screening of women for HPV infection within their models. In total there were five different screening strategies:

- Pap smear test alone (conventional cytology or liquid-based cytology): detection of cervical precancerous lesions
- HPV DNA test alone: detection of HPV infections and HPV types
- Pap test + HPV DNA triage for Pap positive patient
- HPV DNA test + Pap triage for HPV DNA positive patient
- Combined Pap test + HPV DNA test.

Pap test was included as a primary screening measure in all studies including screening, with varying combinations of the inclusion of HPV DNA testing and/or cytological testing as an additional screening measure, or triage.

### Changes to existing cytology-based screening alongside vaccination

#### Liquid-based cytology

As a primary screening strategy, liquid-based cytology was compared to classical cytology in 1 study [29]. Comparing classical cytology and liquid-based cytology in vaccinated women, Goldie et al., 2004 reported a very small increase in QALYs and a marginal raise (between 0.5% and 1.2%) in the reduction in cancer risk with major increase in costs.

#### Changes in screening interval

**Fig. 2:**
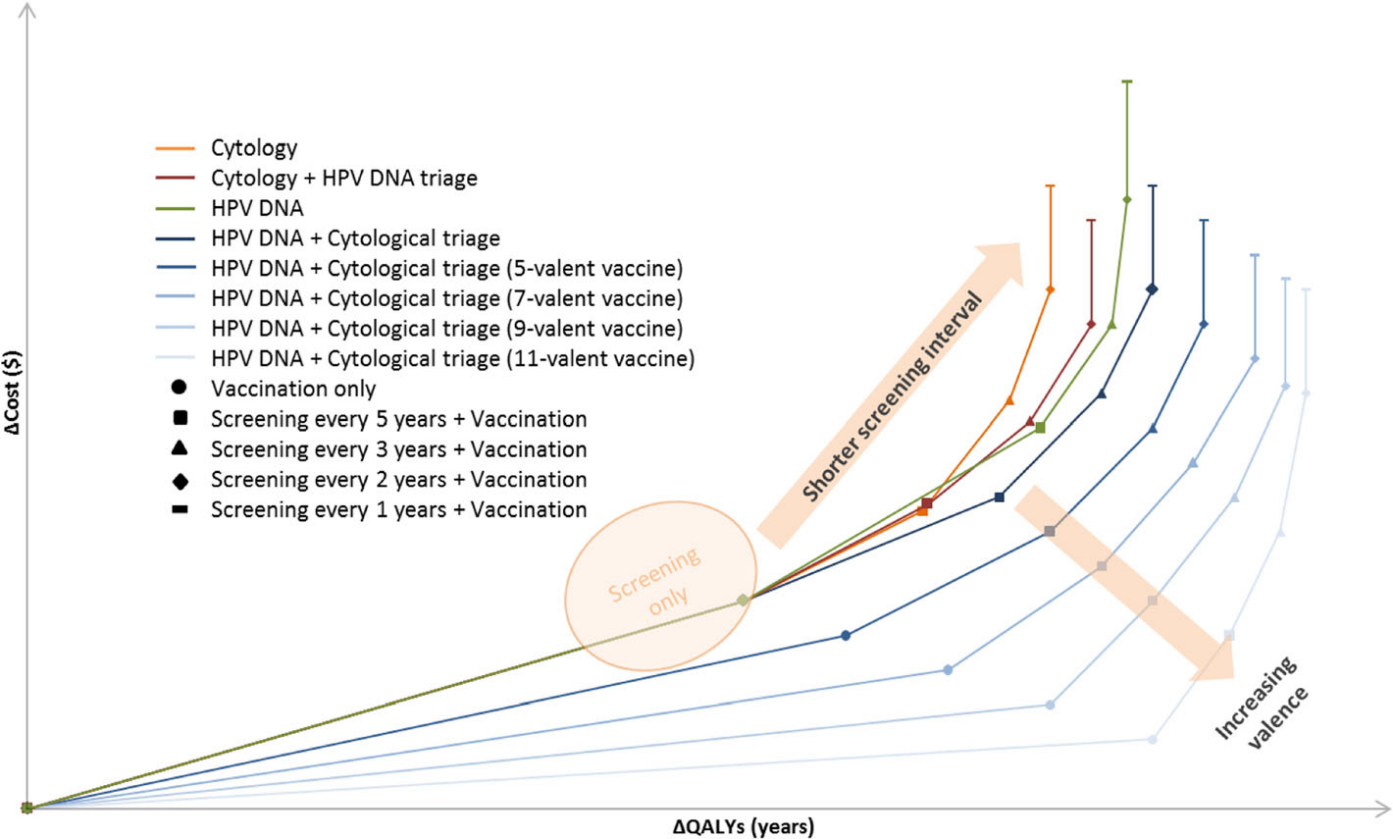
Synthesised results of the systematic literature review. This figure is not to scale, it displays the trend

All studies except one [43] modelled the effect on cost-effectiveness of variation in screening interval in vaccinated persons. Clinical benefit was seen when durations between screenings were reduced; however, this came at an economic cost. By combining vaccination with screening, the screening interval could be increased with only a marginal impact on benefits and a large reduction in costs. Introduction of a vaccine of greater valence was shown to offset negative effects of decreasing screening frequency (see Fig. 2) [37].

#### Delaying screening commencement

Five studies investigated the effect of delaying the age of screening commencement (from ages of 18–35 years old) [27, 29, 33, 35, 39, 40]. Goldie et al., 2004 [29] reported that delaying the age of screening commencement from 21 to 25 years was associated with cost-savings. Delaying the screening age from 30 to 35 was reported not to be cost-effective by Coupe de Melker et al., 2009 and the same trend was reported in Tully et al., 2012 [35, 40].

### Compliance to screening

One study [36] reported the impact of compliance to screening on HPV prevention strategy. A 10% decrease in attendance per screening round for vaccinated women compared to non-vaccinated women, resulted in a marginal impact on cost-effectiveness results.

### HPV DNA test

A strategy of HPV DNA testing was assessed in 5 studies [33, 35, 37, 38, 44] and a combined screening strategy (cytology and HPV DNA testing) in 2 studies [34, 43]. HPV DNA testing triage was assessed in 7 studies [33–35, 38, 42, 43].

Combined screening strategies in combination with vaccination were seen to offer large clinical benefits, at little extra cost in studies including non-conventional techniques when compared to strategies of vaccination only.

After the introduction of HPV vaccination, screening still needed to be continued, and strategies that incorporated HPV testing were more effective and cost-effective than those with cytology alone [43]. strategies involving HPV DNA testing and subsequent cytology triage were associated with a greater QALY gain than a strategy involving vice versa; and, interestingly a strategies of HPV DNA testing alone offered greater QALY gain than those of cytological screening followed by HPV DNA test triage (see Fig. 2).

### Modelling methods

Three modelling approaches were used for assessment in the included studies: 9 studies reported using a standard Markov model [27–29, 35, 36, 38, 39, 41]; 9 studies were based on a standard dynamic model [31–34, 37, 40, 42–44]; and 1 study [30] reported using a two-part model.

## Discussion

### Key findings from literature review

Cervical cancer screening is one of the cornerstones of cervical cancer prevention in association with HPV vaccination. This study explored the cost-effectiveness of alternative HPV prevention strategies that combine screening with vaccination, drawing on 18 publications in order to inform and improve knowledge of the potential impact of the next generation of HPV vaccines.

Among the strategies modelled, HPV DNA testing followed by cytological triage of HPV positive women in combination with HPV vaccination was found to be the optimal strategy, with a comparable cost to other screening strategies and also a greater QALY gain. An increasing vaccine valence counterbalanced the detrimental effects of delayed and less frequent screening (Fig. 2). Strategies with shorter screening intervals were more costly and offered limited added benefit compared to those with longer intervals.

In our review, only 1 study considered changes in screening programs in a context of vaccines that covered than 2 oncogenic HPV types (HPV16/18), with the change in benefits gained from a vaccine of increasing valence offsetting the change in costs associated with a shorter interval between screenings [37]. A nonavalent vaccine, with protection against 5 additional HPV oncogenic types, and 9 HPV types in total, is expected to prevent an even broader spectrum of HPV diseases and in particular to cover from 70% to 90% of cervical cancers. In light of the introduction of this new HPV vaccine to the European markets, a shift in the HPV prevention paradigm is expected, especially in country with high coverage rate.

Simms et al., recently concluded that countries with high vaccination coverage with HPV9 such as Australia and England will require less intensive screening [45]. At this level of protection, the role of screening in vaccinated women will need to be re-examined, and possibly reduced to 3 tests in a lifetime, for example, ages 30, 40, and 60 years; however, this will need to be verified in large studies using a HPV screening test. [46] It is possible that a single screening in combination with HPV9 will produce equivalent results as compared to Gardasil® in combination with 3–4 screenings [47].

One of the main limitations of this review was to exclude models that estimated clinical outcomes alone, instead focusing on economic models estimating clinical and economical outcomes. Nevertheless, economical consideration are rarely disconnected from clinical outcomes in the decision making process. Economic models which make use of the best available data can provide an assessment of the long-term impact of vaccination and screening against HPV and guide decision makers into making a better informed decision regarding which prevention strategy to employ.

The above graph presents a plot of the incremental costs and QALYs of each vaccination strategy with increasing valence. The impact in terms of increasing costs and QALYS on cost-effectiveness results of decreasing the screening interval for each vaccination from no screening to annual screening can then be seen by moving rightwards on each curve. From the above figure, increasing valence is shown to generate additional health benefits at with cost savings versus decreasing the screening interval, which is associated with sharp cost increases for the additional health benefit generated.

### Methodological challenges

Although the conclusions of our review may appear limited because of the time span (until April 2014) and the fact that they do not consider the new nonavalent vaccine, they provide important insights into the methodological challenges in assessing vaccination and screening. Firstly, the choice of the model can limit the scope of the analysis. Most of the models presented in our review are static Markov models and considered only vaccinated women. These models have the advantages of assessing various screening components (different types of screening, change in screening interval, switching age for protocol allowing different tests in younger and older women). Static Markov models cannot adequately take into account herd immunity, age distribution shifts, waning effects, nor do they provide a population-based perspective, therefore do not perfectly reflect disease transmission. In many countries, policy makers will have to consider an existing mix of vaccinated and unvaccinated women.

Static models cannot adequately take into account herd immunity nor age distribution shifts. Risk of infection in susceptible individuals is constant in static models, while in dynamic models, it is a function of the proportion of the population infected (which changes over time). Hence, when intervention uptake is very low (e.g., low vaccine coverage), is targeted at groups that do not have an impact on overall transmission, or does not prevent circulation of the pathogen, static and dynamic models produce similar results.

To assess the change of screening in unvaccinated women, a cohort-based model can be used. Naber et al., 2016 [48] (not included in this review due to timing of publication) recently used an indirect method based on a cohort based-model to investigate at which level of herd immunity screening should be optimised for unvaccinated women. Once herd immunity reached 50%, the authors suggested that reducing screening intensity in both vaccinated and unvaccinated women may be considered, given screening intensity based on pre-vaccination risk levels becomes cost-ineffective [48]. To consider a population-based perspective, 2 types of population-based model co-exist: compartmental dynamic and individual-based dynamic models [31]. Including detailed features of screening such as different test in younger and older women in a compartmental dynamic model is not easy. Individual-based models are by definition more flexible and appear to offer a better combination in terms of prevention strategies modelled and population perspective.

Specific attention needs to be considered to evaluate cervical screening technologies. This was not in the scope of our review but Simonella et al. (2015) [49] demonstrated that the models of organised screening in the evaluation of the cost-effectiveness of HPV vaccination varied in quality. With respect to some important areas of screening (abnormal Pap smear management, diagnostic follow up and management of CIN), models were inconsistent in structure and, in some cases, very simplified. They concluded that models of HPV vaccination can be improved by further attention to the ‘background’ modelling of secondary prevention via cervical screening.

Another difficulty in assessing the best preventive strategies is the number of strategies that can potentially be assessed, and this can quickly become huge if different age intervals, different tests and different algorithms are evaluated. Sander et al. 2016 recently considered 900 combinations of vaccination and screening strategies [50]. The most cost-effective option may depend on the initial set of options considered, with the potential for the comparison of each strategy to a common comparator leading to a sub-optimal decision. In this circumstance it is important to develop an efficiency frontier, removing dominated strategies and those subject to extended dominance; this will lead to the strategy with the highest ICER below the cost-effectiveness threshold being selected.

Regarding the long term impact of HPV vaccines, the scientific community acknowledges that protection against HPV associated diseases related to vaccine types has been demonstrated for at least 10 years and that and long term follow up studies does not show any decrease in efficacy over time [51]. In addition, recent WHO guidance on cervical cancer the duration and strength of effectiveness of cross protection is still to be demonstrated [52]. Therefore scenarios with cross-protection and or with a low duration of protection could be considered as less relevant and were therefore omitted from this analysis.

### Next steps

A single programme of vaccination and screening will need to be deliberated when considering further reduction of the cervical cancer burden [17, 53]. Additionally, introduction of HPV DNA testing in several European countries is seen as having the potential to alter the cervical cancer screening paradigm. There is still considerable uncertainty around the direction of this change and further research is needed in order to assess the impact on the cost-effectiveness profile of HPV prevention strategies. It will be important to obtain high coverage through vaccination, meaning screening will be needed less regularly; however, a challenge will remain to accurately identify those who have been vaccinated, as this will occur prior to the need for screening.

Some governments, such as in Australia, have already adapted their screening programme, accelerating the implementation of HPV DNA testing, due to the success of vaccination [53]. Moreover, the availability of a nonavalent HPV vaccine will enable a complete review of current cervical cancer prevention strategies, in both a primary and secondary setting, offering the opportunity for a more efficient and affordable approach.

Existing analyses in this article, and the recent ones not included in our review [48, 50], focus on a 3-doses HPV vaccine and/or a vaccine that protects against 70% of cervical cancer and are probably obsolete [48]. New analyses considering changes in screening programs in a context of a nonavalent vaccine that protects against 90% of cervical cancers and using more realistic vaccination programmes in term of costs (2 dose schedule instead of 3 dose) and coverage rate (vaccinated and unvaccinated population) are needed.

As referred to by Mendes et al., 2015 the choice of optimum cervical cancer screening strategies will be highly complex due to the number of criteria to consider from a policy viewpoint and especially relevant in countries with high vaccination coverage rates [54].

Further research on country-specific data for HPV vaccination and screening as well is the corresponding economic impact should be conducted to generate evidence which can assist policy-makers in finding a more systematic and tailored approaches to HPV prevention.

## Conclusion

This review has highlighted how HPV prevention strategies have been demonstrated to show both an economic and epidemiological impact. The arrival of a new HPV vaccine has the potential to dramatically alter the epidemiological outlook of HPV, and, as a consequence, current screening programmes may need to be rethought. The need to re-assess current prevention programmes is increasingly highlighted [55].

This review has demonstrated synergies between screening and vaccination. New prevention strategies involving multi-valence vaccination, HPV DNA test screening, delayed commencement and frequency of screening could be implemented in the future.

HPV prevention strategies implemented in the future should be chosen with care, and informed knowledge of the potential impact of all possible prevention strategies. Availability of a nonavalent vaccine will allow a complete review of current strategies, offering an opportunity for a more efficient and affordable approach to HPV prevention.

Also highlighted in this review is the difficulty in assessing the interaction between screening and vaccination and in assessing multiple strategies in general. Appropriate modelling techniques will need to be utilised to assess the most cost-effective strategies, with recommendations made based on analysis of efficiency frontiers and similar techniques.

## Appendix 1

### Systematic review search methods

**Table 4:** PICOS framework

PICOS	Definition
Population	All individuals
Intervention	Vaccination against HPV infection, with a cervical screening strategy
Comparators	One of the following three scenarios: - Vaccination against HPV infection, with an alternative cervical screening strategy - Vaccination against HPV infection only - An alternative cervical screening strategy only
Outcomes	Health economic endpoint (with a focus on the cost-effectiveness result (quality-adjusted life-year, cost-effectiveness ratio) and clinical outcomes (cancers/cases avoided)
Study types	Cost-effectiveness analyses

**Table 5:** Exclusion criteria

Exclusion criteria	Notes
Null entries	No information is reported in title and abstract fields
Duplicates	Duplicate of an existing entry
Not in the language of interest	English only
Abstract that is reported elsewhere	A conference abstract with the content reported in another publication
Not study type of interest	Not a cost-effectiveness analysis
Not HPV vaccination	Not including an arm of HPV vaccination ± screening
Not the country of interest	Countries of interest include: Austria, Belgium, Switzerland, Czech Republic, Germany, Denmark, Spain, Finland, France, Greece, Ireland, Italy, the Netherlands, Norway, Poland, Portugal, Sweden, Slovenia, and the UK, the US, Canada and Australia
No outcome of interest	Health economic endpoints

## Appendix 2

### Systematic review results

**Table 6:** Overview of included studies – base case (sensitivity analyses) presented

	Kulasingam 2003 [27]	Sanders 2003 [28]	Goldie 2004 [29]	Taira 2004 [32]	Kulasingam 2007 [39]
Country	US	US	US	US	Australia
Modelling approach	Markov Model	Markov Model	Markov Model	Deterministic transmission Model	Markov Model
Disease included	HPV, CIN1,2–3, CC	HPV,SIL, CC	HPV, CIN1,2–3, CC	HPV	HPV, CIN1,2–3, CC
*HPV types (High Risk/ Low Risk)*	yes/yes	16,18,31,33,35,39,45,51,52,56,58,59,68/20,22	16,18,non 16–18/yes	16,18/-	16,18,non 16–18/yes
Target population	12 F to 85 F	12 F	13 F to ≈ 17 F	12 F to 50 F	12 F to 85 F
Time horizon	12 to 85	12 to Lifetime	12 to Lifetime	12 to Lifetime	12 to 85
Discount rates	3%	3% (0–5%)	3%	2% (0–5%)	5% (3–5%)
Perspective	Societal	Societal	Societal	Societal	Societal
	Goldhaber-Fiebert 2008 [33]	Kim 2008 [31]	Coupe, de Melker 2009 [35]	Coupe, van Ginkel 2009 [36]	Kim 2009 [30]
Country	US	US	Netherlands	Netherlands	US
Modelling approach	Individual-based model	Dynamic Model	Markov Model	Markov Model	Hybrid model (transmission model + disease model)
Disease included	HPV, CIN1,2–3, CC	CIN, CC, other HPV-related disease	HPV, CIN1,2–3, CC	HPV, CIN1,2–3, CC	CIN, CC, other HPV-related diseases
*HPV type (High Risk/Low Risk)*	16,18,non 16–18/yes	16,18/-	16,18,31,33,35,39,45,51,52,56,58,59,68/-	16,18,31,33,35,39,45,51,52,56, 58,59,66,68/-	16,18/-
Target population	9 F to Lifetime	12 F	12 F	12 F	12 F + M
Time horizon	9 to Lifetime	12 to Lifetime	12 to Lifetime	12 to Lifetime	12 to Lifetime
Discount rates	3%	3%	4% (Costs) 1.5% (Health)	4% (Costs) 1.5% (Health)	3%
Perspective	Societal	Societal	Societal	Societal	Societal
	Kim, Ortendahl 2009 [34]	Accetta 2010 [38]	Diaz 2010 [43]	Demarteau 2011 [41]	Burger 2012 [42]
Country	US	Italy	Spain	France	Norway
Modelling approach	Individual-based Model	Markov Model	Micro-simulation model	Markov Model	Simulation model
Disease included	HPV, CIN1,2–3, CC	HPV, CIN1,2–3, CC	HPV, CIN1,2–3, CC	HPV, CIN1,2–3, CC	HPV, CIN1,2–3, CC
*HPV type (High Risk/Low Risk)*	16,18/-	16,18,non 16–18/yes	16,18,non 16–18/yes	-	16,18,non 16–18/yes
Target population	35–45 F	F	11–14 F	12 F	F
Time horizon	35–45 to Lifetime	11 to Lifetime	11 to lifetime	12 to lifetime	Lifetime
Discount rates	3%	3%	3%	3% (Costs) 1.5% (Health) (0–5%)	4%
Perspective	Societal	Societal	Societal	Societal	Societal
	Coupe 2012 [37]	Tully 2012 [40]	Berkhof 2013 [44]		
Country	Netherlands	Canada	Slovenia, Poland		
Modelling approach	Individual-based model	Transmission model	Individual-based model		
Disease included	HPV, CIN1,2–3, CC	HPV, CIN1,2–3, CC	HPV, CIN1,2–3, CC		
*HPV type (High Risk/Low Risk)*	16,18,31,33,35,39,45,51,52,56, 58,59,66,68/-	16,18,non 16–18/-	16,18,−/−		
Target population	10 F	18 F	12 F		
Time horizon	10 to Lifetime	80 years	Not clear		
Discount rates	4% (Costs) 1.5% (Health) (No-3%)	3%	3%		
Perspective*	Societal	Societal	Societal		

*Societal perspective – costs and QALYs are calculated based on health forgone as a result of costs falling on the healthcare budget and displacing other healthcare activities

**Table 7:** Prevention strategies

	Kulasingam 2003 [27]	Sanders 2003 [28]	Goldie 2004 [29]	Taira 2004 [32]	Kulasingam 2007 [39]
Strategy assessed	.No Intervention .Vaccination only (12 F) .Cytology 18 F - 5y . Cytology 18 F - 3y . Cytology 18 F - 2y . Cytology 18 F - 1y .Vaccination (12 F) + Cytology 30 F - 5y .Vaccination (12 F) + Cytology 18 F - 5y .Vaccination (12 F) + Cytology 26 F - 3y .Vaccination (12 F) + Cytology 18 F - 3y .Vaccination (12 F) + Cytology 24 F - 2y .Vaccination (12 F) + Cytology 18 F - 2y .Vaccination (12 F) + Cytology 22 F - 1y .Vaccination (12 F) + Cytology 18 F - 1y	.Cytology (16 F) - 2y (.Vaccination (12 F) + Cytology (16 F) - 1y) .Vaccination (12 F) + Cytology (16 F) - 2y (.Vaccination (12 F) + Cytology (16 F) - 3y .Vaccination (12 F) + Cytology (16 F) - 4y .Vaccination (12 F) + Cytology (16 F) - 5y)	.No intervention .Cytology 35 F - 5y .Cytology 30 F - 5y .Cytology 25 F - 5y .Vaccination (12 F) + Cytology 35 F - 5y .Vaccination (12 F) + Cytology 30 F - 5y .Vaccination (12 F) + Cytology 25 F - 5y .Vaccination (12 F) + Cytology 21 F - 5y .Vaccination (12 F) + Cytology 25 F - 3y .Vaccination (12 F) + Cytology 21 F - 3y .Vaccination (12 F) + Cytology 21 F - 2y .Vaccination (12 F) + Cytology 18 F - 2y .Vaccination (12 F) + Cytology LBC 18 F - 2y .Vaccination (12 F) + Cytology 18 F - 1y .Vaccination (12 F) + Cytology LBC 18 F - 1y	. Cytology ?F - 2y (.Vaccination (12 F) + Cytology ?F - 4y .Vaccination (12 F) + Cytology ?F - 3y)) .Vaccination (12 F) + Cytology ?F - 2y (.Vaccination (12 F) + Cytology ?F - 1y)	.No intervention .Vaccination only (12 F) .Cytology 18-21 F - 2y .Vaccination (12 F) + Cytology 18-21 F - 2y .Vaccination (12 F) + Cytology 18-21 F - 3y .Vaccination (12 F) + Cytology 25 F - 3y
Results	ICER/life year gained, reduction in cervical cancer incidence and mortality	ICER/QALY gained, reduction in cervical cancer incidence and mortality	ICER/QALY gained, reduction in lifetime risk of cervical cancer	ICER/QALY gained	ICER/life year gained
	Goldhaber-Fiebert 2008 [33]	Kim 2008 [31]	Coupe, de Melker 2009 [35]	Coupe, van Ginkel 2009 [36]	Kim 2009 [30]
Strategy assessed	.No intervention .Vaccination only (9 F) .Cytology 18 F,21 F,25 F – 5y,3y,2y,1y + HPV triage (Switch 25y,30y,35y) .HPV 18 F - 1y + Cytology triage .Vaccination (9 F) + Cytology 18 F,21 F,25 F – 5y,3y,2y,1y + HPV triage (Switch none, 25y,30y,35y) .Vaccination (9 F) + HPV 25 F - 3y + Cytology triage	.Cytology 20 F - 1y .Cytology 20 F - 2y .Cytology 20 F - 3y + HPV Triage .Vaccination (12 F) + Cytology 20 F - 1y .Vaccination (12 F) + Cytology 20 F - 2y .Vaccination (12 F) + Cytology 20 F - 3y + HPV Triage	.Vaccination only (12 F) .Cytology 30 F,35 F - 4,5,6,7 rounds .Cytology + HPV triage 30 F,35 F - 4,5,6,7 rounds .HPV + Cytology triage 30 F,35 F - 4,5,6,7 rounds .Cytology & HPV 30 F,35 F - 4,5,6,7 rounds	.Cytology 30 F - 5y .Vaccination (12 F) + Cytology 30 F - 5y	.Cytology 25 F/M - 3y + HVP triage .Vaccination (12 F) + Cytology 25 F/M - 3y + HVP triage .Cytology 25 F/M - 2y + HVP triage .Vaccination 12 F/M + Cytology 25 F/M - 3y + HVP triage .Vaccination 12 F + Cytology 25 F/M - 2y + HVP triage .Vaccination 12 F/M + Cytology 25 F/M - 2y + HVP triage
Results	ICER/QALY gained, reductions in cervical cancer	ICER/QALY gained, reductions in cervical cancer	ICER/QALY gained, cervical cancer cases and cervical cancer deaths avoid	ICER/QALY gained, cervical cancer cases and cervical cancer deaths avoid	ICER/QALY gained,
	Kim, Ortendahl 2009 [34]	Accetta 2010 [38]	Diaz 2010 [43]	Demarteau 2011 [41]	Burger 2012 [42]
Strategy assessed	.Cytology + HPV triage lifetime for 35 F every 1y, 2y, 3y, 4y .Cytology + HPV triage + Combined cytology/HPV after 35 F every 1y, 2y, 3y, 4y .Cytology + HPV triage lifetime for 45 F every 1y, 2y, 3y, 4y .Cytology + HPV triage + Combined cytology/HPV after 45 F every 1y, 2y, 3y, 4y	.No intervention .Cytology 25 F - 3y .Cytology 25 F - 5y .HPV DNA 25 F - 3y .HPV DNA 25 F - 5y .Cytology 25 F - 3y + HPV triage .Cytology 25 F - 5y + HPV triage .HPV DNA 25 F - 3y + Pap Triage .HPV DNA 25 F - 5y + Pap Triage .Vaccination only (11 F) .Vaccination (11 F) + Cytology 25 F - 3y .Vaccination (11 F) + Cytology 25 F - 5y .Vaccination (11 F) + HPV DNA 25 F - 3y .Vaccination (11 F) + HPV DNA 25 F - 5y .Vaccination (11 F) + Cytology 25 F - 3y + HPV triage .Vaccination (11 F) + Cytology 25 F - 5y + HPV triage .Vaccination (11 F) + HPV DNA 25 F - 3y + Pap Triage .Vaccination (11 F) + HPV DNA 25 F - 5y + Pap Triage	.Cytology alone .Cytology with HPV triage .Combined cytology and HPV .Vaccination + cytology alone .Vaccination + cytology with HPV triage .Vaccination + combined cytology and HPV	.Cytology ?F – 3y .Vaccination (12 F) + Cytology ?F – 3y (.Cytology ?F – 1y .Vaccination (12 F) + Cytology ?F – 1y .Cytology ?F – 5y .Vaccination (12 F) + Cytology ?F – 5y)	.No vaccinated .Vaccinated only (12 F) .Screening frequency 3y to 6y, wait time for rescreen 6 m or 12 m, additional HPV+/Cyt- 1 to 3
Results	ICER/QALY gained, reductions in lifetime risk of cervical cancer	ICER/life year gained, lifetime risk of cervical cancer, reduction in cancer risk, cervical cancer mortality	ICER/life year gained, reduction in cervical cancer	ICER/QALY gained	ICER/life year gained, reduction in cancer
	Coupe 2012 [37]	Tully 2012 [40]	Berkhof 2013 [44]		
Strategy assessed	.Cytology 30 F - 5y .Vaccination only .Vaccination + Cytology 30 F-7 times .Vaccination + Cytology 30 F-6 times .Vaccination + Cytology 30 F-5 times .Vaccination + Cytology 30 F-4 times .Vaccination + HPV DNA screening-7 times + cytological triage .Vaccination + HPV DNA screening-6 times + cytological triage .Vaccination + HPV DNA screening-5 times + cytological triage .Vaccination + HPV DNA screening-4 times + cytological triage	.Vaccination (12 F) + Cytology 21 F .Vaccination (12 F) + Cytology 25 F	.Vaccination only (12 F) .10,6,3-yearly Pap .10,6,3-yearly HPV .Vaccination (12 F) +10-yearly Pap .Vaccination (12 F) +10-yearly HPV .Vaccination (12 F) +6-yearly Pap .Vaccination (12 F) +6-yearly HPV		
Results	ICER/QALY gained, cancer cases and deaths	ICER/QALY gained, ICER/life year gained	ICER/QALY gained		

## Abbreviations

CHMP: Committee for medicinal products for human use
CIN: Cervical intra-epithelial neoplasia
DNA: Deoxyribonucleic acid
EMA: European medicines agency
EU: European union
FDA: Food and drug administration
HPV: Human papilloma virus
ICER: Incremental cost-effectiveness ratio
LYG: Life years gained
NHS: National health service
QALY: Quality-adjust life-year
SCC: Squamous-cell carcinoma
YLS: Years of life saved
